# On the origin of nitrosylated hemoglobin in COVID-19: Endothelial NO capture or redox conversion of nitrite? Experimental results and a cautionary note on challenges in translational research

**DOI:** 10.1016/j.redox.2022.102362

**Published:** 2022-06-09

**Authors:** Renato C. Nogueira, Magdalena Minnion, Anna D. Clark, Alex Dyson, José E. Tanus-Santos, Martin Feelisch

**Affiliations:** a Department of Pharmacology, Ribeirao Preto Medical School, University of São Paulo, Brazil; b Clinical and Experimental Sciences, Faculty of Medicine, University of Southampton, UK; c Southampton NIHR Biomedical Research Centre, University Hospital Southampton NHS Foundation Trust, UK; d Centre for Pharmaceutical Medicine Research, Institute of Pharmaceutical Science, King’s College London, London, SE1 9NH, UK

**Keywords:** Nitric oxide, Nitrite, Oxidative stress, Thiols, Ascorbate, Hemoglobin

## Abstract

In blood, the majority of endothelial nitric oxide (NO) is scavenged by oxyhemoglobin, forming nitrate while a small part reacts with dissolved oxygen to nitrite; another fraction may bind to deoxyhemoglobin to generate nitrosylhemoglobin (HbNO) and/or react with a free cysteine to form a nitrosothiol. Circulating nitrite concentrations in healthy individuals are 200-700 nM, and can be even lower in patients with endothelial dysfunction. Those levels are similar to HbNO concentrations ([HbNO]) recently reported, whereby EPR-derived erythrocytic [HbNO] was lower in COVID-19 patients compared to uninfected subjects with similar cardiovascular risk load. We caution the values reported may not reflect true (patho)physiological concentrations but rather originate from complex chemical interactions of endogenous nitrite with hemoglobin and ascorbate/N-acetylcysteine. Using an orthogonal detection method, we find baseline [HbNO] to be in the single-digit nanomolar range; moreover, we find that these antioxidants, added to blood collection tubes to prevent degradation, artificially generate HbNO. Since circulating nitrite also varies with lifestyle, dietary habit and oral bacterial flora, [HbNO] may not reflect endothelial activity alone. Thus, its use as early marker of NO-dependent endothelial dysfunction to stratify COVID-19 patient risk may be premature. Moreover, oxidative stress not only impairs NO formation/bioavailability, but also shifts the chemical landscape into which NO is released, affecting its downstream metabolism. This compromises the endothelium’s role as gatekeeper of tissue nutrient supply and modulator of blood cell function, challenging the body’s ability to maintain redox balance. Further studies are warranted to clarify whether the nature of vascular dysfunction in COVID-19 is solely of endothelial nature or also includes altered erythrocyte function.

## Highlights

• Nitrosylhemoglobin (HbNO) has recently been suggested as marker of endothelial dysfunction in COVID-19.
• Blood was processed with N-acetylcysteine + ascorbate to enable detection of endogenous HbNO.
• We find that addition of NAC + Asc to blood gives rise to artefactual HbNO formation when nitrite is present.
• Our results suggest this method requires further optimisation before it can be used for stratification of patient risk.

## 1 Introduction

Nitric oxide (NO) formation by vascular endothelial cells underlies endothelium-dependent vasorelaxation, modulates blood cell function and numerous other regulatory processes [1,2]. NO released into the vascular lumen is metabolised via four principal routes: the majority is scavenged by reaction with oxyhemoglobin in erythrocytes, forming nitrate (NO_3_^-^) and methemoglobin. A small fraction escapes to react with dissolved oxygen, forming nitrite (NO_2_^-^). Another part may react with deoxyhemoglobin to form HbNO and/or with a reactive cysteine (Cys-ß93) to form a nitrosothiol (SNOHb). Reported [HbNO] in human blood ranges from undetectable [[3], [4], [5], [6]] to micromolar [7]. After the discovery of SNOHb in 1996 [8], concentrations and relevance of this species have been subject to a lively debate [3,[9], [10], [11]]. Both heme products are mechanistically linked via a proposed NO group transfer from HbNO to SNOHb via an enigmatic thiol-mediated process [7].

Circulating nitrite concentrations in healthy humans (200–700 nM) vary with lifestyle and dietary intake. Concentrations progressively decrease with increasing cardiovascular risk load [12]. Those levels are curiously close to the [HbNO] recently reported by Montiel et al. [13]; using EPR spectroscopy, these authors measured lower erythrocytic [HbNO] in COVID-19 patients compared to uninfected subjects with similar cardiovascular risk. Together with elevated plasma concentrations of lipid (per)oxides and lower concentrations of NOx (nitrite + nitrate) they interpreted this signature as indicative of endothelial dysfunction secondary to an oxidative stress-induced decrease in NO bioavailability. These results are in line with earlier reports [14] of a decreased availability of NO (and hydrogen sulfide, H_2_S) metabolites in patients with COVID-19, although that study did not specifically measure HbNO. We here demonstrate that the [HbNO] reported by Montiel et al. [13] likely originated from chemical interaction of endogenous nitrite with (partially deoxygenated) hemoglobin and ascorbic acid/N-acetylcysteine (Asc/NAC), and may not reflect true (patho)physiological levels. Using a recently refined method to quantify [HbNO] by ferricyanide-induced one-electron oxidation and detection of released NO by gas-phase chemiluminescence we show that addition of these antioxidants to blood does not prevent HbNO decomposition but indeed generates the very species the investigators intended to preserve.

## 2 Methods

### 2.1 Blood collection and processing

Our study received local (University of Southampton’s Ethics and Research Governance Online Nr. 30507.A2) and national (Health Research Authority, IRAS project ID: 236117/SA1) ethics approval. Venous blood was obtained from healthy human participants using EDTA-vacutainer tubes (Becton Dickinson) and a 21G butterfly needle. Red blood cells (RBCs) from 15 individuals were collected before the pandemic, processed as described below, and kept frozen at -80°C until analysis. Experiments with fresh blood were conducted in March 2022 following further method refinement. The antioxidant mixture (NAC and l-Asc; 5 mM final concentration) or an equivalent volume of vehicle was added to blood immediately before centrifugation from a 10x stock in 100 mM phosphate buffer pH 7.4. Following gentle mixing by inversion, blood was centrifuged immediately (5,000×*g* for 2min, or 800×*g* for 10 or 20min at room temperature (RT)). Supernatant (plasma) and buffy coat (platelets and white cells) were rapidly removed, and RBCs either used directly or snap-frozen in liquid nitrogen and stored at -80°C. We recommend minimising blood exposure to air to avoid raising oxygen saturation levels, leading to artificial sample ‘arterialisation’. Frozen RBC pellets were thawed on crushed ice and analysed immediately.

### 2.2 Hemoglobin and HbNO preparation

Oxyhemoglobin (HbO_2_) was prepared from crystallized human hemoglobin [15]. Fully and partially deoxygenated hemoglobin were prepared by adding sodium dithionite to HbO_2_ or passing a gentle stream of argon over the HbO_2_ stock solution surface for 20min. Standards of partially nitrosylated hemoglobin species (HbNO/HbO_2_ hybrids; 1, 10, and 25% heme occupancy) were prepared by adding substoichiometric quantities of sodium nitrite to dithionite-containing hemoglobin in phosphate buffer under argon, followed by size exclusion chromatography (PD10 columns, Merck-Sigma) in ambient air. These solutions are relatively stable (t_1/2_ 0.5-2 h) when kept at RT in the dark. Fully nitrosylated (tetranitrosylhemoglobin; Hb_4_[NO]_4_) was prepared by exposing deoxyHb to a 20% molar excess of the NO-donor MAHMA/NONOate (Calbiochem) in oxygen-free phosphate buffer (100 mM, pH 7.4), followed by size-exclusion chromatography; this standard was less stable (t_1/2_ 15–20 min at RT) under aerobic conditions.

### 2.3 HbNO and nitrite measurements

Our original method for HbNO quantification used oxidative sample processing in combination with gas-phase chemiluminescence NO detection [16,17]. Here, the sample gas inlet of the detector is connected to a reaction chamber containing 50 mM potassium ferricyanide in phosphate-buffered saline (PBS), maintained at 37°C and continuously purged with argon (110 mL/min); antifoam SE-15 is added to minimise foaming. NO is released from HbNO following oxidation of the heme ‘underneath the ligand’ (ferric heme has a considerably lower affinity for NO than its reduced form), and HbNO is quantified by integrating the area under the curve for gas-phase NO, measured by a specific chemiluminescence detector (CLD88sp, EcoMedics, Duernten, Switzerland).

**Fig. 1:**
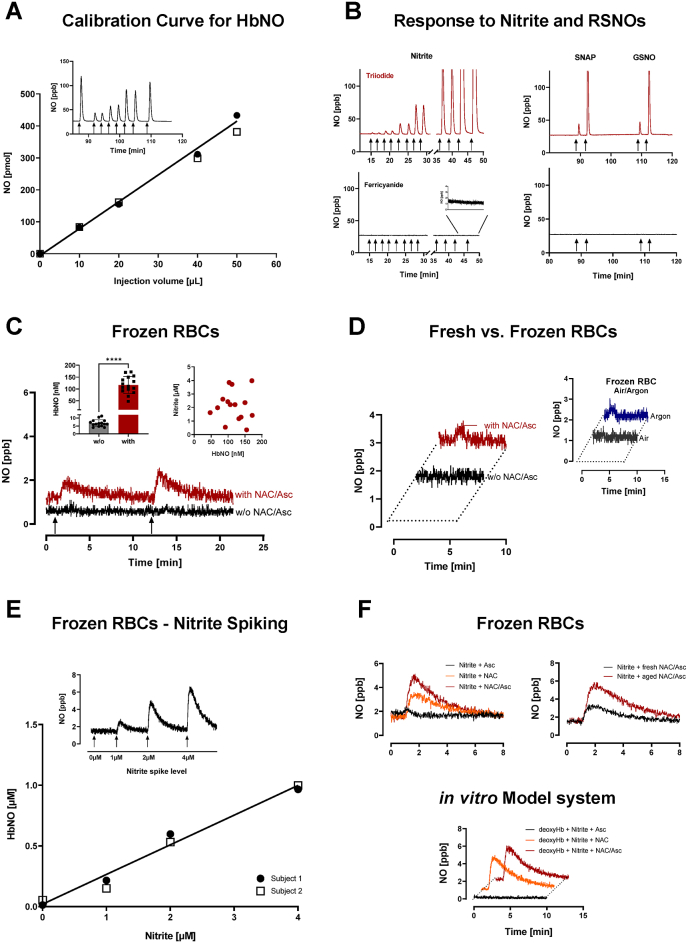
Response characteristics of the ferricyanide-based HbNO assay and representative experimental results on the chemical interaction of nitrite, N-acetylcysteine (NAC), ascorbic acid (Asc) and hemoglobin in intact human red blood cells (RBCs) and a simple *in vitro* model system. ***A) Linearity in detector response to increasing amounts of HbNO injected into ferricyanide-containing reaction solution.*** Exemplary result from routine calibrations with freshly produced standards. *Inset:* Corresponding original NO response depicting duplicate injections, bracketed by 50 μL of a diluted HbNO stock (note the minor difference between first and last peak, indicative of HbNO decomposition).***B) Lack of cross-reactivity to nitrite and S-nitrosothiols (RSNOs).*** Control experiments were conducted in parallel using two separate reaction chambers and scrubbers, each connected to their own chemiluminescence detector (CLD88 sp), one chamber containing triiodide in acetic acid (upper panels) and the other aqueous ferricyanide solution (lower panels). *Left panels*: responses to increasing concentrations of nitrite (duplicate injections of 0.1, 0.3, 1, 3, 10, 100 μM; *Inset:* response to 10 and 100 μM nitrite at higher magnification); *Right panels*: single injections of 1 and 10 μM S-nitroso-N-acetylpenicillamine (SNAP) and S-nitrosoglutathione (GSNO). Arrows indicate times at which analytes were injected.**C) *Addition of NAC + Asc to RBC pellets previously collected in the absence of these antioxidants gives rise to HbNO formation.*** Representative recordings of chemiluminescence signals for NO obtained upon injection of aliquots of stored erythrocytes from two human subjects with and without (w/o) NAC/Asc; RBC pellets were diluted 1:20 in phosphate buffer pH 7.40, and 500 μL aliquots were injected into reaction chambers (arrows). *Left inset*: Data from RBCs of 15 human subjects (25–60 ys of age) without (w/o) and with addition of NAC/Asc demonstrate considerable inter-individual variation with values ranging from 3.9 to 11.3 nM (6.8 ± 2.1 nM) at baseline and 47.2 to 171.3 nM (116.6 ±36.4 nM) in the presence of NAC/Asc (****p<0.0001). *Right inset:* Lack of correlation between endogenous nitrite and detected HbNO concentrations. These experiments demonstrate that NAC/Asc addition does not preserve decomposition of a labile species but promotes HbNO formation from a preformed compound not detected by this assay.**D) HbNO detection in RBCs of freshly drawn blood using the standard (800×*g*, 10 min) centrifugation protocol, in the absence and presence of NAC + Asc, and effects of partial deoxygenation.***Left panel:* Representative recording from experiments with RBCs of three study participants; signal intensity was similar when using the fast centrifugation protocol (not shown). Adding NAC/Asc to fresh RBCs produces a slightly bigger signal when added to whole blood before centrifugation. *Right panel:* Change in HbNO signal from frozen RBCs (collected without added antioxidants) following partial deairation before injection. Thus, addition of antioxidants (NAC/Asc) before centrifugation also promotes HbNO formation in fresh blood, and the ability to detect a signal in the absence of antioxidants is enhanced by partial removal of oxygen.**E) In the presence of NAC/Asc, spiking of RBCs with nitrite increases HbNO formation.** Aliquots of frozen and subsequently deairated RBC pellets were incubated with increasing concentrations of nitrite for 5 min before injection into the reaction chamber. Representative results from separate experiments with RBCs from two different subjects. *Inset:* Representative original recoding from identical cell numbers incubated with increasing nitrite concentrations. HbNO formation is dependent on the presence of nitrite and increases in a concentration-dependent manner.**F) HbNO is also formed in a model system comprising deoxyhemoglobin (deoxyHb), NAC, Asc and nitrite.***Upper left panel:* Frozen and subsequently deairated red blood cells (RBCs) were incubated with 5 mM NAC, Asc, alone or in combination, before injection into the reaction chamber. *Upper right panel:* Apparent HbNO concentrations are higher using “aged” NAC (i.e. when stock solutions were exposed to air for a longer time). *Lower panel):* HbNO production from partially deoxygenated hemoglobin solutions and nitrite in the presence of NAC and Asc in phosphate buffer pH 7.40. These results demonstrate that HbNO generation does not require red blood cell constituents other than hemoglobin, a thiol and nitrite and is dependent on redox status, with ascorbate making a minor contribution to the overall signal in the absence of cells. (For interpretation of the references to colour in this figure legend, the reader is referred to the Web version of this article.)

We modified the original method (50 mM ferricyanide in PBS, 37°C) for use with human RBCs and found increased sensitivity with reactor solutions kept at 60°C and substitution of PBS for Milli-Q water (adjusted to pH 7.4). The latter permits direct injection of intact RBCs into the hypotonic reaction solution, omitting the need to prepare cell lysates. The elevated reaction temperature, more rapid cell lysis, and heme oxidation with fast NO-release kinetics facilitate quantification of lower [HbNO]. Detector responses to freshly prepared HbNO solutions are linear over a wide concentration range, and neither nitrite nor S-nitrosothiols (RSNOs) produce a signal in this assay (see Fig. 1A/B). Detection limits depend on total cell number, but are typically ≤10 nM at injection volumes of 0.5 mL using RBC suspensions. In some experiments with frozen RBCs, concentrations of HbNO and nitrite were determined in parallel; for the latter, RBCs were lysed 1:4 in MilliQ water, deproteinised by addition of 75% (v/v) methanol, and immediately measured using a dedicated analysis system (ENO-20, EiCom).

### 2.4 Incubations

All incubations were carried out with room air-equilibrated or argon-gassed phosphate buffer (pH 7.4, 100 mM), as appropriate. Fresh or frozen RBCs were diluted 1:20 in phosphate buffer, incubated for 5 min at RT ± Asc/NAC (5 mM final concentrations; 225 mOsm/kg). In some experiments, RBC pellets were deaerated by passing a gentle stream of argon over their surface before dispersion in deoxygenated phosphate buffer. HbO_2_ in phosphate buffer was kept in a septum-sealed reaction vial either at room air or under argon following partial deoxygenation to mimic venous blood oxygenation levels. All experiments were conducted at RT.

## 3 Results and discussion

During refinement of our techniques for detection and quantification of nitrogen and sulfur related metabolites in biological samples, we found that addition of thiols and other reducing compounds to blood can alter the concentration of certain analytes and give rise to artificial HbNO formation in erythrocytes. A synopsis of our results with N-acetylcysteine and ascorbate as adjuvants for HbNO detection is depicted in Fig. 1. In frozen RBCs from healthy human subjects, physiological steady-state [HbNO] are in the single-digit nanomolar range, independent of whether RBCs were harvested using conventional centrifugation protocols (10–20 min, 800×*g*) or processed within 3 min after blood collection (2 min at 5000×*g*). These levels rise to >100 nM upon addition of Asc/NAC, with marked inter-individual variability (Fig. 1C). We obtained similar results in fresh blood, with enhanced signal intensity following partial deoxygenation (Fig. 1D). Despite a lack of association between endogenous nitrite and [HbNO] levels (Fig. 1C, *inset*) RBC spiking with nitrite increased [HbNO] in a concentration-dependent manner (Fig. 1E). The results obtained with fresh and frozen RBCs could be reproduced using a simple *in vitro* model system comprising deoxyHb, NAC, Asc and nitrite; a greater increase in signal intensity observed with older stock solutions of NAC (undergoing autoxidation) suggests redox status modulates the interaction of nitrite with hemoglobin (Fig. 1F). The latter may explain the lack of association between endogenous nitrite and [HbNO] we observed in frozen RBCs (Fig. 1C). Collectively, our data demonstrates that the previously documented lack of detection of HbNO in human blood [[3], [4], [5], [6]] is not due to the perceived instability of this analyte but rather that its concentration is below quantifiable limits unless nitrite is subjected to redox transformation by additional adjuvants.

Since circulating nitrite is not only dependent on endothelial NO production but also on dietary nitrate intake and composition/activity of the oral mucosal flora [18], the [HbNO] detected in COVID patients [13] may not necessarily be a reflection of endothelial activity alone (even in the absence of adjuvants used to stabilise this species). The exact mechanism through which HbNO is generated under these conditions is unclear, but there is precedence for numerous reactions (beyond NO capture by deoxyHb) in the literature. Protonation of nitrite (e.g. in the stomach) forms nitrous acid (HNO_2_) which decomposes to generate NO in a reaction that is enhanced by reducing compounds such as ascorbic acid [19]. Deoxyhemoglobin itself can also reduce nitrite to NO [18,20], albeit rather inefficiently. At physiological pH and partial pressure of oxygen *in vivo*, nitrite is biotransformed, in a thiol and heme-dependent manner, to nitrosothiols and NO-heme species [21]. Elsewhere, concomitant formation of S-nitrosocysteine and NO-myoglobin was first explored in the context of nitrite’s effects on colour formation and prevention of bacterial growth in meat 5 decades ago [22]. Thiolate anions (RS^-^) interact with nitrosothiols to release nitroxyl (NO^-^/HNO) [23], which can react with methemoglobin to form HbNO [24]. Thus, in the presence of thiols, Asc and heme HbNO formation from nitrite is complex and can occur via multiple interdependent routes. This makes reaction outcomes difficult to predict and cautions against using additional factors that can influence the redox environment without full optimisation and assessment of their impact on analyte concentrations.

The difficulties other groups experienced in detecting [HbNO] in human blood at baseline [[3], [4], [5], [6]] are likely due to methodology-inherent limitations in sensitivity. Although the main concern we here raise relates to sample pretreatment and is method independent, other pifalls with the method used by Montiel and colleagues [13] deserve further consideration. NO bound to the α-subunits of Hb has a characteristic signature with a triplet hyperfine structure readily detected by EPR; however, sensitivity is limited [25], not least due to a large signal from plasma/RBC-bound ceruloplasmin (and possibly superoxide dismutase) masking the much smaller heme-nitrosyl signal. Those challenges can be overcome either by spectral deconvolution applying regression analysis or by subtraction methods using multiple sample aliquots. Using the former approach, which does not require any sample pretreatment, a detection limit of ∼200 nM for HbNO has been achieved [6]. Although no signal was detected in arterial or venous human blood at baseline, levels robustly increased to micromolar levels upon NO inhalation [6]. Balligand’s group uses an approach that involves digital subtraction of two large EPR signals from the composite spectrum (and adjustment) to obtain a small one [26,27]. It assumes that the free radical (and the copper) EPR signals do not change shape when blood is exposed to ascorbate and/or N-acetylcysteine. This is questionable given the ease with which metals react with ascorbate and thiols [28,29]), which may explain the “paradoxical effects” Balligand’s group observe with Asc in about half of their subjects (in which case they use ‘reverse subtraction’ [26]). In order to obtain a pure ‘free radical signal’ the authors expose another RBC aliquot for 30min to air; this will produce metHb, which in addition to a large signal at g = 6 also has a small one in the g = 2 region that may interfere with the subtraction method. Moreover, the dissociation rate of NO from penta-coordinate α-nitrosyl Hb [30] gives a lifetime of 46 min, not dissimilar to the lifetime of HbNO/oxyHb hybrids we prepare as standards; thus, a 30min exposure of RBCs to air would leave substantial HbNO (notwithstanding the possible presence of other nitrosylhemoglobin signals besides the penta-coordinate α-nitrosyl). Despite these reservations, the similarity in [HbNO] detected after addition of Asc/NAC by ourselves and Balligand’s group [13] is remarkable given the orthogonal principles of detection and dramatically different temperatures (-196°C versus +60°C) used. However, the basal [HbNO] we measured in the absence of any adjuvants are substantially lower than those reported by other groups including Balligand’s [7,13].

These considerations do not call into question the overall importance of the COVID study we have questioned, since both circulating nitrite and NO levels are decreased in patients with endothelial dysfunction [12]. In health, venous nitrite levels are similar in plasma and RBCs. The use of fasting plasma nitrite concentration as biochemical marker of endothelial activity is still not universally accepted, despite strong experimental support using stable-isotope labelling and NOS-stimulation/inhibition in animals (including eNOS-knockout mice) and humans [12,31,32]. Crucially however, as Fig. 1C clearly demonstrates, [HbNO] does not correlate with intracellular nitrite levels; it is additionally influenced by oxygenation and redox status, as shown earlier using NO-donors [33,34], and possibly other factors such as CO_2_/bicarbonate concentrations [35]. Thus, the suggested use of [HbNO] measurements as early marker of NO-dependent endothelial dysfunction [13] may be premature and should be confirmed following further exploration of sample processing and assay optimisation. Once the mechanisms of formation are better understood it could inform new nutritional and/or pharmacological approaches for the care of patients with COVID-19 (and possibly other viral infectious diseases).

The enhanced oxidative stress that accompanies viral infections not only impairs NO availability to regulate blood flow/pressure, but also shifts the chemical landscape into which NO is released [36]. This has important consequences for enzymatic NO production, the endothelium as gatekeeper of nutrient supply to tissues, and the modulation of blood cells other than platelets. The multi-system nature of COVID-19 also imposes a formidable challenge to achieving redox balance at the whole-organism level [36]. Irrespective of the methods used to quantify different NO-species, dedicated studies are thus required to further characterise these processes and identify whether the nature of vascular dysfunction in COVID-19 is chiefly due to endothelial perturbation or extends to altered erythrocyte function [37,38].

Translational research faces many challenges. Whilst experimental animal models have greatly improved our understanding of (patho)physiology and pharmacology, specific kinetics and mechanisms of reactions can differ between rodents and humans, often considerably. This is particularly pertinent for metal and thiol-based chemistries, and hemoglobin-related reactions represent a case in point. The SNO-Hb story is a stark reminder of these analytical and conceptual challenges; >25years following its discovery [8] we are still awaiting the final verdict [39,40], notwithstanding substantial animal and human experimentation [10,11,41]. Both species differences and the complexity of human physiology underline the importance of experimental medicine in humans. The challenge of understanding the myriad interconnected and interdependent sensing and adaptation processes that preserve homeostasis in the face of ongoing environmental/metabolic challenges becomes even more pertinent when considering the systematic study of specific signaling pathways in complex redox disease processes such as COVID-19 [36]. Indeed, wherever redox reactions are involved, outcomes tend to become less predictable, despite electron exchange processes underpinning the origin of Life and subsequent evolution of human physiology [42,43].

The above should serve to reinforce the need for active dialogue across the many fields that redox biology impacts upon. Peer-reviewed publication sits at the heart of this as a forum for honest and open discussion, and as a proponent of scientific rigour. Implicit in this is the willingness to acknowledge and correct mistakes, in the pursuit of scientific truth. This pertains to investigators, journal editors and readers alike, and not doing so or wilfully ignoring the uncomfortable only perpetuates error, further increases the high costs of research and obstructs scientific progress. While in science truth will always prevail, structures in place that support its dissemination currently leave little opportunity for rebuttal or reappraisal. In translational medicine, misinformation can translate into poor outcomes – perhaps here the need for change is more urgent than in other disciplines. Does this mean measuring HbNO after addition of an antioxidant cocktail should not be published? Absolutely not, but we ought to be clear that those numbers do not represent true *ex vivo* concentrations, and until we understand what processes they actually reflect, we currently caution against the use of HbNO for risk stratification in COVID-19.

## 4 Contributions

MF conceived the study and wrote the manuscript. RCN, MM and ADC conducted experiments, acquired and verified data; AD and JETS provided important intellectual content. All authors critically reviewed and revised the manuscript.

## Declaration of competing interest

The authors have no conflict of interest to declare.
